# Work-related slip, trip and fall injuries reported by National Health Service staff in Great Britain: how many are due to slipping?

**DOI:** 10.1136/ip-2023-045210

**Published:** 2024-07-23

**Authors:** Mark Liddle, Gillian Nicholls, David Leigh, Jennifer Kinder, Alison Curran, Michael Zand

**Affiliations:** 1 HSE Science and Research Centre, Health and Safety Executive, Buxton, UK; 2 Health and Safety Executive, Bootle, UK

**Keywords:** Occupational injury, Registry, Workplace

## Abstract

**Background:**

Workplace injuries due to a slip, trip or fall on the level (STF) are often reported together, making the potential impact of targeted interventions, such as slip-resistant footwear, difficult to assess. The objective of this research was to review workplace non-fatal injuries reported as STFs under the Reporting of Injuries, Diseases and Dangerous Occurrences Regulations 2013 to determine what proportion of staff STF injuries reported by the National Health Service (NHS) in Great Britain were caused specifically by a slip.

**Methods:**

The free text descriptions of all 1004 STF injuries reported by NHS staff in summer 2018 and winter 2018/2019 were independently reviewed by two researchers to determine whether a slip was the primary cause or not. Where agreement could not be reached or the cause was unclear, an STF specialist reviewed the reports to establish the likely cause. The kappa statistic was used to measure inter-reviewer agreement, and the χ^2^ test was used to compare proportions across seasons.

**Results:**

The reviewers agreed on the initiating event, slip or non-slip, for 917 (91.3%) of the incidents. The kappa statistic was 0.842 (95% CI 0.785 to 0.898) indicating strong agreement between reviewers. In total, 431 or 42.9% (95% CI 39.8% to 46.1%) of the STF incidents were slips. This percentage was greater in winter compared with summer (49.0% and 36.0%, respectively, p<0.001).

**Conclusion:**

The high proportion of slips among reported STF injuries implies that an effective intervention targeting workplace slips in the NHS could have a substantial impact on the number of injuries reported.

**WHAT IS ALREADY KNOWN ON THIS TOPIC**

**WHAT THIS STUDY ADDS**

- Slips accounted for about 43% of the reported staff slip, trip and fall injuries within Great Britain’s National Health Service in 2018/2019.

**HOW THIS STUDY MIGHT AFFECT RESEARCH, PRACTICE OR POLICY**

- The large proportion of slips among reported slip, trip and fall injuries helps to judge the possible impact that targeted and effective interventions, such as the provision of appropriately specified slip-resistant footwear, may have on the number of reported incidents. This, in turn, helps to identify reasonably practicable interventions for duty holders to manage the risk of workplace slips, trips and falls.

## Introduction

Slips, trips and falls on the level (STF), together, are the most common kind of workplace injury reported in Great Britain (GB). In 2022/2023, they accounted for 32% (just over 19 000) of the non-fatal employee injuries reported to GB’s Health and Safety Executive (HSE).^1^ Although reported together, the cause of an STF injury can vary depending on the initiating event and, hence, have different mitigation measures—for example, regular cleaning of floor contamination can help to prevent slips but will have little impact on trips.

Two recent randomised controlled trials have evidenced the potential for appropriate slip-resistant footwear to reduce workplace slips.^2 3^ In particular, one undertaken by GB’s National Health Service (NHS) found the provision of appropriate slip-resistant footwear could reduce workplace slips among NHS staff by about 37%.^3^ To understand the significance of these findings and the potential impact of effective targeted workplace interventions if implemented by duty holders, it is important to understand the contribution of slips specifically. This study, therefore, aims to estimate the proportion of reported workplace STF injuries within the NHS caused by a slip.

## Methods

The NHS is the overarching term used to refer to the four publicly funded healthcare systems in the UK for each of the devolved nations (England, Wales, Scotland and Northern Ireland). It currently directly employs around 1.3 million full-time equivalent (FTE) staff in England, 158 000 FTE in Scotland, 95 000 FTE in Wales and 65 000 FTE in Northern Ireland.^4^ This includes nurses, health visitors, doctors, ambulance professionals and NHS support staff; the figures do not include most staff working in primary care, which includes general practitioners and dentists, since they tend not to be directly employed by the NHS.^4^

In GB (comprising England, Wales and Scotland), the Reporting of Injuries, Diseases and Dangerous Occurrences Regulations 2013 (RIDDOR 2013)^5^ puts duties on employers, some self-employed and people in control of work premises to report certain serious workplace injuries, occupational diseases and specified dangerous occurrences (‘near misses’).^5^ If a worker is injured while at work, then the incident is reportable under RIDDOR 2013 if it results in: death; a specified injury; or they are unable to carry out their normal duties for more than seven consecutive days following the day of the incident.^6^ For injuries due to STFs, specified injuries are generally fractures, with other over-7-day injuries mainly resulting in sprains or contusions. Some injuries to non-workers that occur on work premises are also reportable under RIDDOR, but these are out of the scope of this work. Incidents are reported to HSE by the employer, self-employed or person in control of the work premises. Each report is then considered in line with HSE’s incident selection criteria by the relevant enforcing authority, usually HSE or the relevant local authority (LA). For this study, which focused on incidents occurring in the NHS, nearly all premises are expected to be enforced by HSE and not LAs.

This study considered non-fatal incidents reported: between June 2018 and May 2019; as an STF; and with ‘nhs’ in the email address of the notifier. This would capture STF incidents in GB involving workers employed both directly and indirectly by the NHS. Injuries to members of the public were excluded. This identified 1941 STF incidents.

A previous review of work-related STF injuries in the USA and Sweden found slipperiness or slipping contributed to around 40–50% of fall-related injuries^7^; sample size calculations showed that if 40% of the STF RIDDOR reports were slip-related, a sample size of 1000 would produce a two-sided 95% CI for the estimated proportion with a width equal to 6.2 percentage points.^8^ Including all reports over the three months of summer (June, July and August) and the three months of winter (December, January and February) provided a sample of the size of this order (n=1004), and ensured coverage of the two extremes in season where the percentage of slips could vary.

Each RIDDOR 2013 report contains a free-text narrative of the incident. This narrative is provided by the notifier, and they determine what text details to provide. This study only uses that narrative text, and no additional information is used. A pilot was undertaken where two reviewers were asked to review the first 100 incidents. The two reviewers were not STF specialists and had no specialist training to undertake the review; although both had familiarity with RIDDOR 2013 reports through previous work. For each incident, they reviewed the free-text description to assess whether a slip was the primary cause, something else or whether it was unclear; an incident therefore could be designated a slip, non-slip or unclear. The reviewers agreed on 91 of the 100 incidents, and there was disagreement or the text was unclear for 9 incidents. Overall, the reviewers were comfortable with the task and so proceeded with the remaining incidents following the same methodology. Once all 1004 incidents had been independently reviewed, an STF specialist reviewed the incidents where there was disagreement or the assignment was unclear to provide the final assessment.

Results are summarised in total and by season. The kappa statistic was used as a measure of inter-reviewer agreement, exact binomial CIs were used for the proportion of incidents that were slips, non-slips or unclear and the χ^2^ test was used to compare proportions across seasons. All analyses were conducted in Microsoft Excel and Stata.^9^

### Patient and public involvement

This work was part of the Stopping Slips among Healthcare Workers trial, which consulted NHS staff (aged 20–71 years) from diverse roles including nurses, catering, housekeeping and doctors about: the rationale for the trial; shoe styles; use of text messages; follow-up duration; and testing slip-resistance of usual work footwear. An NHS employee was a member of the joint Trial Steering Committee and Data Monitoring and Ethics Committee.

## Results

Table 1 shows the results of the review. The reviewers agreed on the initiating event, slip or non-slip, for 917 (91.3%) incidents. The kappa statistic was 0.842 (95% CI 0.785 to 0.898) indicating strong agreement between reviewers. STF specialist input was required on 87 (8.7%) incidents. There were 467 (46.5%) incidents in summer and 537 (53.5%) in winter. In total, 431 or 42.9% (95% CI 39.8% to 46.1%) of the STF incidents were slips. The percentage of STF incidents that were slips was statistically significantly higher in winter compared with summer (49.0% and 36.0%, respectively, χ^2^(1)=17.2, p<0.001). The STF specialist noted that 5 of the 87 incidents they reviewed probably should not have been reported as an STF since the free-text suggested some other incident type. For this exercise, these incidents were included in the non-slip totals (table 1).

**Table 1:** Results of the review of 1004 workplace non-fatal injuries reported as a slip, trip or fall on the same level in Great Britain during summer 2018 and winter 2018/2019

	Season		Total
	Summer 2018 (June, July and August)	Winter 2018/2019 (December, January and February)	
Reviewed, N	467	537	1004
Agreement*, N (%)	426 (91.2)	491 (91.4)	917 (91.3)
Specialist review, N (%)	41 (8.8)	46 (8.6)	87 (8.7)
			
Slips			
N	168	263	431
% (95% CI)^†^	36.0 (31.6 to 40.5)	49.0 (44.7 to 53.3)	42.9 (39.8 to 46.1)
Non-slips			
N	287	261^‡^	548^‡^
% (95% CI)^†^	61.5 (56.9 to 65.9)	48.6 (44.3 to 52.9)	54.6 (51.4 to 57.7)
Unclear			
N	12	13	25
% (95% CI) ^†^	2.6 (1.3 to 4.4)	2.4 (1.3 to 4.1)	2.5 (1.6 to 3.7)

* Excludes three incidents where the agreement was ‘unclear’.

† Exact binomial 95% CI.

‡ Includes five incidents identified as potentially not a slip, trip or fall on the level during the specialist review.

## Discussion

This study found that slips represented about 43% of the staff STF injuries reported by the NHS in 2018/2019. This is slightly lower than the proportion observed in USA healthcare (56%^10^ and 55%^11^) but comparable with results from the USA and Sweden that covered all sectors (40–50%^7^). The latter suggests the current results could be broadly indicative of all sectors in GB. However, it would be beneficial to replicate this review for other sectors or time frames in GB, to see how this proportion may differ. In any future reviews, it would be helpful to breakdown non-slips further to identify trips and other types of falls separately, and to consider other characteristics, for example, whether the incident occurred outside or inside. It would also be beneficial to explore STF proportions among different demographics. Automated text mining techniques would make this process more efficient if reliable and accurate methods can be found.

The current review found there was a higher proportion of slips during the winter months than in the summer months (49% and 36%, respectively). This finding may not be surprising but is still noteworthy as the risk of trips may also be influenced by factors such as weather conditions, seasonal work and shorter daylight hours. It should be noted the reviewers were aware of the season when coding and so may have been unintentionally biased toward classifying an incident as a slip during the winter months.

Information provided in the RIDDOR reports was not always clear, which may have led to the misclassification of some incidents. However, the high reviewer agreement and a small number of ‘unclear’ incidents provide confidence in the classification assigned. The review also revealed incidents that should not have been classified as an STF. In this case, the number was small and so would not greatly impact results. However, there could be STF injuries misclassified as another injury type and therefore not included. The scale and potential impact of this misclassification is unknown.

Overall, the high proportion of slips among reported staff STF injuries suggests an effective intervention, if implemented by duty holders to target workplace slips in the NHS, could have a substantial impact on the number of injuries reported. Repeating this exercise in other sectors would help to determine the importance of slips as a proportion of STF injuries in other industries.
